# Ultrastructural Characterization of Membrane Rearrangements Induced by Porcine Epidemic Diarrhea Virus Infection

**DOI:** 10.3390/v9090251

**Published:** 2017-09-05

**Authors:** Xingdong Zhou, Yingying Cong, Tineke Veenendaal, Judith Klumperman, Dongfang Shi, Muriel Mari, Fulvio Reggiori

**Affiliations:** 1 Department of Preventive Veterinary Medicine, College of Veterinary Medicine, Northeast Agricultural University, Harbin 150030, China; x.zhou02@umcg.nl; 2 Department of Cell Biology, University of Groningen, University Medical Center Groningen, A. Deusinglaan 1, 9713 AV Groningen, The Netherlands; y.cong@umcg.nl; 3 Department of Cell Biology, Center for Molecular Medicine, University Medical Center Utrecht, Heidelberglaan 100, 3584 CX Utrecht, The Netherlands; A.Veenendaal-3@umcutrecht.nl (T.V.); j.klumperman@umcutrecht.nl (J.K.)

**Keywords:** PEDV, *alpha-coronavirus*, lifecycle, electron microscopy, membrane rearrangement

## Abstract

The porcine epidemic diarrhea virus (PEDV) is a *coronavirus* (*CoV*) belonging to the *α-CoV* genus and it causes high mortality in infected sucking piglets, resulting in substantial losses in the farming industry. *CoV* trigger a drastic reorganization of host cell membranes to promote their replication and egression, but a detailed description of the intracellular remodeling induced by PEDV is still missing. In this study, we examined qualitatively and quantitatively, using electron microscopy, the intracellular membrane reorganization induced by PEDV over the course of an infection. With our ultrastructural approach, we reveal that, as most of *CoV*, PEDV initially forms double-membrane vesicles (DMVs) and convoluted membranes (CMs), which probably serve as replication/transcription platforms. Interestingly, we also found that viral particles start to form almost simultaneously in both the endoplasmic reticulum and the large virion-containing vacuoles (LVCVs), which are compartments originating from the Golgi, confirming that *α-CoV* assemble indistinguishably in two different organelles of the secretory pathway. Moreover, PEDV virons appear to have an immature and a mature form, similar to another *α-CoV* the transmissible gastroenteritis coronavirus (TGEV). Altogether, our study underlies the similarities and differences between the lifecycle of *α-CoV* and that of viruses belonging to other *CoV* subfamilies.

## 1. Introduction

*Coronaviruses* (*CoV*) are enveloped positive single-stranded RNA viruses that are characterized by crown-like spikes on the virion surface under an electron microscope [1]. Mostly based on phylogenetic clustering, this virus family has been divided into four subgroups: the *α-*, *β-*, *γ-*, and *Δ-CoV* [1,2]. The porcine epidemic diarrhea virus (PEDV) is an *α-CoV* and the causative agent of porcine epidemic diarrhea, which is characterized by severe enteritis, vomiting, watery diarrhea, and loss of weight. PEDV infections have a substantial detrimental effect on the swine industry because they cause high morbidity and mortality rates, especially in sucking piglets [3,4,5]. Since its first identification in Belgium in 1978 in growing and fattening pigs [6], PEDV has been reported in Europe and Asia, and a remarkable increase of PEDV outbreaks occurred in the pig-producing provinces of China in late 2010 [7]. PEDV also emerged for the first time in the United States in early 2013 [5], and spread to Canada and Mexico [8]. These recent outbreaks and the global re-emergence of PEDV have attracted the attention of numerous virologists, representing the necessity for urgent attention and a deeper understanding of PEDV biology and mechanisms of pathogenesis.

Replication is a fundamental event in the lifecycle of viruses, and in the case of positive single-strand RNA viruses and some double-stranded DNA viruses, it occurs in cellular compartments that are generated by specialized viral proteins through the modification of one or more host membranes and/or organelles [9,10,11]. *CoV*-infected cells also undergo a massive remodeling of intracellular membranes [12]. Upon *CoV* entry in the host cell and release of their genomic RNA (gRNA) into the cytoplasm, two large polyproteins known as pp1a and pp1b, are synthesized, and their self-processing leads to the generation of 15 to 16 non-structural proteins (nsp) [13,14]. Nsp proteins trigger the formation of double-membrane vesicles (DMVs) and convoluted membranes (CMs), which provide a platform for the concentration of viral factors to very likely guarantee the efficient replication and transcription of *CoV* gRNA [12,15,16,17,18]. Collectively, the nsp proteins also form replication-transcription complexes (RTCs), which localize on the DMVs and CMs where they mediate the synthesis of viral RNA [15,19,20]. Double-stranded RNA (dsRNA), a byproduct of genomic RNA replication, becomes concentrated in the lumen of the DMVs through a mechanism that remains totally unknown [15]. In contrast, virion assembly takes place at the endoplasmic reticulum (ER)-Golgi intermediate compartment (ERGIC) and Golgi complex, and involves the inward budding of the limiting membrane of these compartments, which is triggered by the interaction between the nucleocapsid (N) protein associated with the gRNA, and the structural spike (S), membrane (M), and envelope (E) proteins [13,14]. Complete virions subsequently reach the extracellular environment following the conventional secretory pathway [21].

The first electron microscopy (EM) analyses of *CoV*-infected cells were carried out in 1960s, and mainly characterized the morphology of the viral particles [16,17,18,22]. Subsequent ultrastructural studies highlighted the DMVs as being a feature of *CoV* infections, including those of mouse hepatitis virus (MHV), severe acute respiratory syndrome coronavirus (SARS-CoV), human CoV NL63 (HCoV-NL63), Middle East respiratory syndrome coronavirus (MERS-CoV), and infectious bronchitis virus (IBV) [12,15,23,24,25,26]. Over the years, other intracellular structures have been reported to be present in cells infected by *CoV* in addition to DMVs and virion particles, including CMs, tubular bodies (TBs), vesicle packages (VPs), cubic membrane structures (CMSs), and large viron-containing vacuoles (LVCVs). CMs are reticular inclusions observed in between clusters of DMVs, and are often connected with both DMVs and the ER as revealed by both 2D and 3D ultrastructural studies [15,17]. Like DMVs, MHV- and SARS-CoV-induced CMs are positive for dsRNA and nsp proteins as well, and this finding has led to the postulation that these structures are also involved in viral replication and transcription [12,15,27,28]. In later stages of SARS-CoV infection, groups of single-membrane vesicles surrounded by a common outer membrane, called VPs, arise from the merging of DMVs, viron particles, and possibly CMs [15,27]. LVCVs, which are vacuoles filled with viral particles, have been frequently observed in cells infected with *CoV*; they are Golgi cisternae that expand to accommodate the increasing assembly of progeny virions over the course of an infection, as they are positive for Golgi marker proteins [12,17,18,22,23,26,27,29,30]. Finally, TBs and CMSs, which have been detected in MHV- and SARS-CoV-infected cells, are condensed, highly organized membrane rearrangements connected to the ER. TBs and CMSs appear to be the result of an aggregation of an overproduced protein because they mostly contain a single structural protein, and therefore they are probably a byproduct of a massive infection with no role in virus replication [12,17,31,32].

Despite its veterinary relevance, the intracellular membrane remodeling induced in host cells by PEDV remains largely unknown. The only infections of *α-CoV* characterized at the ultrastructural level so far are those of HCoV-NL36 and of transmissible gastroenteritis coronavirus (TGEV) [23,29,33]. In cells infected with these two *α-CoV*, virions were observed in the ER, Golgi, and LVCVs, whereas DMVs were only reported to be present in cells exposed to HCoV-NL36. We thus decided to analyze PEDV-infected cells by quantitative EM and immuno-electron microscopy (IEM) to establish a more comprehensive inventory of the membrane remodeling induced by this virus and in more general by *α-CoV*. We opted for a time-course approach to determine at which stage of the viral lifecycle the different membranous structures appear. By combining the ultrastructural data with the measurement of viral RNA synthesis, viral replication, progeny virus release, and immunofluorescence analyses, our results show that PEDV infection induces a profound reorganization of the ER and Golgi, which precedes the formation of DMVs, CMs, and LVCVs. Consistently with these observations, we also found that the ER and Golgi undergo alterations not observed for other *CoV*, such as ER proliferation and a Golgi vacuolarization, which results in an organelle that we have named the irregular vesicle clusters (IVCs). Importantly, and similar to HCoV-NL36 and TGEV, we also found that together with the Golgi, the ER is the major platform of virion assembly. Altogether, our study provides an overall comprehensive picture of the ultrastructural events taking place inside a cell over the course of a PEDV infection.

## 2. Materials and Methods

### 2.1. Cell Culture, Virus Propagation, and Time-Course Analysis of PEDV Infection

Vero E6 cells, a kindly gift from Jolanda Smit [34,35], were maintained with Dulbecco’s Modified Eagle Medium (DMEM, Gibco, Waltham, MA, USA) containing 5% fetal calf serum (FCS, Gibco). The used PEDV strain was CV777, and it was grown as previously described in Vero E6 cells [36].

Vero E6 cells were also employed for the time-course infection [36,37]. Vero E6 cells at 60% confluence were inoculated with one multiplicity of infection (MOI) of PEDV and 25 µg/mL of trypsin (Sigma-Aldrich, St. Louis, MO, USA). After 1 h of incubation, cells were washed with phosphate-buffered saline (PBS, 137 mM NaCl, 2.7 mM KCl, 10 mM Na_2_HPO_4_, 1.8 mM KH_2_PO_4_, pH 7.4) to remove the excess virus and to synchronize the PEDV lifecycle. Infected cells were maintained in complete DMEM without FCS, and aliquots of cells and culture supernatants were collected for analysis at 0, 8, 16, 24, 36, 48, 60, and 72 h post-infection (p.i.).

### 2.2. Extracellular Virus Titration

The amount of infectious PEDV particles released into culture medium was determined by calculating the 50% tissue culture infectious (TCID_50_) values in Vero E6 cells. Briefly, monolayers of Vero E6 cells were inoculated with serial dilutions in DMEM of supernatants collected at 0, 8, 16, 24, 36, 48, 60, and 72 h p.i. Cytopathic effects, i.e., cell death, were morphologically assessed under a light microscope after incubation at 37 °C for 4 days. The TCID_50_ value was calculated using the Spearman/Kaerber formula [38,39].

### 2.3. Isolation of the Total RNA and Real-Time PCR (RT-PCR)

Quantification of viral replication was determined by RT-PCR. Briefly, the total RNA was extracted from cells using TRIzol (Invitrogen, Carlsbad, CA, USA) according to the manufacturer’s instructions. First-strand cDNA was synthesized by using the Moloney murine leukemia virus (M-MLV) reverse transcriptase and oligo (dT) (both reagents were from Invitrogen). RT-PCR was then performed using primers to detect the mRNA levels of the ORF3 (GAGACTCGAGCGATTGACACAGTTG and GAGAGGTACCGCCTCAAAGAAGACGC) and the N protein (GAGACTCGAGCGTCTGAAAAGCCAATC and GAGAGGTACCGGTTATTGCCTCTGTTG) in a CFX Connect™ Real-Time PCR detection system (Bio-Rad, Hercules, CA, USA) using the following conditions: 10 s at 95 °C, then 40 cycles of 5 s at 95 °C, and finally 34 s at 60 °C.

### 2.4. Immunofluorescence Analyses

Immunofluorescence analyses were carried out as previously described [12,40]. Preparations were first incubated with the primary antibodies in PBS containing 0.1% bovine serum albumin (BSA) at room temperature for 1 h and, after extensive washing with PBS, they were incubated with the secondary antibodies also in PBS containing 1% BSA at room temperature for 45 min. Preparations were finally mounted in 4′,6-diamidino-2-phenylindole (DAPI)-containing ProLongTM Gold antifade (Invitrogen). Fluorescence signals were captured with either a Leica TCS SP8 confocal microscope (Leica Microsystems, Wetzlar, Germany) or a DeltaVision Elite system (GE Healthcare Life Sciences, Pittsburgh, PA, USA).

Primary antibodies used in this study were monoclonal antibodies against dsRNA (English and Scientific Consulting Kft, Szirák, Hungary), TGN46 (Sigma) and PDI (Enzo, Farmingdale, NY, USA), and polyclonal antisera against GIANTIN (Abcam, Cambridge, UK) and GM130 (Abcam). Secondary antibodies were Alexa488- or Alexa568-conjugated goat anti-rabbit or anti-mouse antibodies (Life Technologies, Waltham, MA, USA).

### 2.5. Transmission Electron Microscopy

Cells were fixed with Karnovsky (2% para-formaldehyde (PFA), 2.5% glutaraldehyde (GA) in 0.1 M sodium cacodylate pH 7.4) for 140 min at room temperature and then post-fixed with 1% OsO_4_, 1% KCNFe in 0.1 M sodium cacodylate buffer (pH 7.4) for 1 h on ice. Samples were subsequently dehydrated stepwise with increasing concentrations of ethanol before rinsing them with 1,2-propylene oxide (Merck, Darmstadt, Germany) at room temperature and embedding them in Epon resin [41]. After resin polymerization for 4 days at 60 °C, 65–70 nm sections were cut using an UC7 ultra-microtome (Leica Microsystems) and contrasted with uranyl acetate and lead citrate [41], before being analyzed in a CM100bio TEM (FEI, Eindhoven, The Netherlands).

For the quantitative analyses, at least 200 cell profiles were randomly selected at each p.i. timepoint, and three different quantifications were performed on four grids. First, the number of cell sections containing at least one of the PEDV-induced structures was counted to morphologically determine the proportion of infected cells. Second, we calculated the percentage of cell sections positive for each PEDV-induced structure. Third, we determined the average number of each structure per cell section and calculated the standard deviation. The average diameter of the length of each structure was determined by measuring 10–40 profiles using the ImageJ software (http://rsb.info.nih.gov/ij/), which also calculated the standard deviations. At each p.i. timepoint, the ER surface area per cell was calculated using the point-hit method [42] on 50 randomly selected electron micrographs from three different grids. The results are presented relative to the 0 h p.i. control.

### 2.6. Immuno-Electron Microscopy

Cells were fixed in 4% PFA in 0.1 M phosphate buffer (19 mM NaH_2_PO_4_, 81 mM Na_2_HPO_4_, pH 7.4) overnight at 4 °C. Before scraping them from the petri dish in PBS containing 1% gelatin, cells were washed three times in PBS and one time in PBS containing 50 mM glycine. Cells were successively embedded in 12% gelatin, cryo-sectioned, and immunogold labelled as previously described [43]. Primary antibodies were mouse anti-KDEL (Calbiochem, San Diego, CA, USA), sheep anti-TGN-46 (Serotec, Oxford, UK) mouse anti-GM130 (BD Biosciences, Franklin Lakes, NJ, USA), mouse anti-CD63 (Developmental Studies Hybridoma Bank, Iowa City, IA, USA), and mouse anti-transferrin receptor (TfR, ThermoFisher Scientific, Waltham, MA, USA), and they were detected after bridging with a rabbit anti-mouse IgG (Rockland, Limerick, PA, USA) or an rabbit anti-sheep IgG antibody (Nordic, Tady, Sweden), respectively, with 10-nm gold particles conjugated to protein A (CMC, Utrecht, The Netherlands). Labelled cryo-sections were contrasted with a 2% uranyl oxalate (pH 7) and methyl cellulose-uranyl acetate (pH 4) solution on ice, and imaged as described for the conventional EM.

## 3. Results

### 3.1. PEDV Induces the Formation of Multiple Membranous Structures

**Figure 1:**
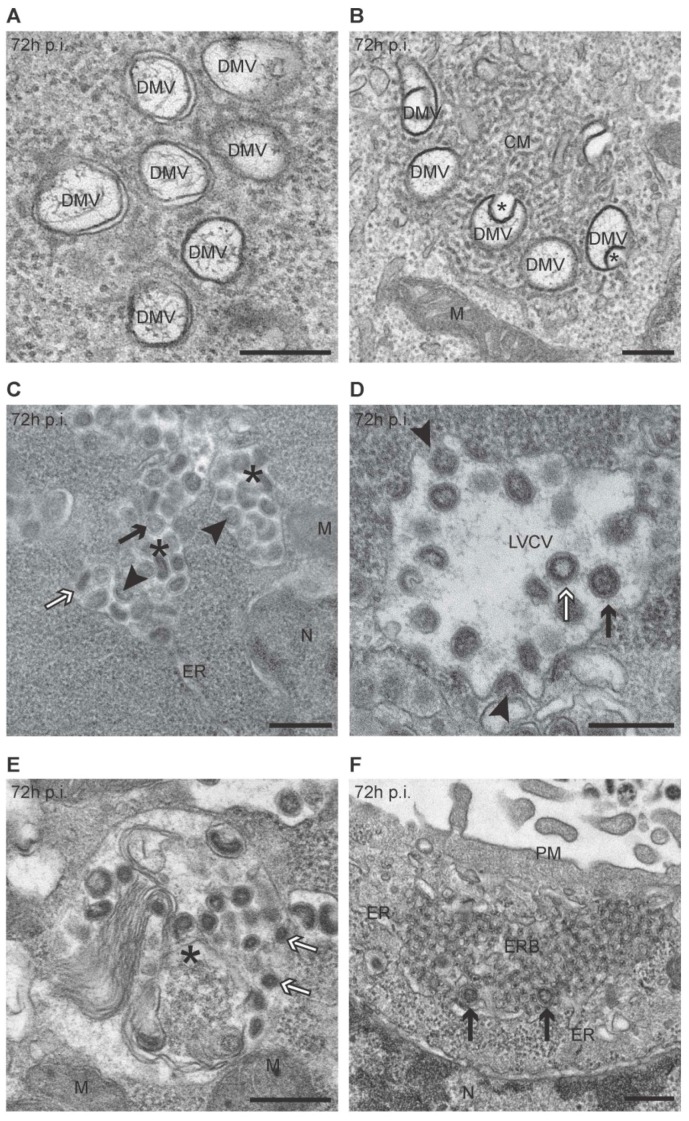
Porcine epidemic diarrhea virus (PEDV) induces the formation of multiple membranous structures. Vero E6 cells were inoculated with PEDV (multiplicity of infection (MOI) = 1) and processed for electron microscopy (EM) at 72 h p.i. as described in the Materials and Methods section. (**A**,**B**) Representative electrographs of double-membrane vesicles (DMVs) and convoluted membranes (CMs) are shown. Asterisks indicate invaginations of the DMVs limiting membrane; (**C**) expanded ER with luminal virions (asterisks). Arrowheads indicate forming viral particles. Black and white arrows indicate immature and mature virions, respectively; (**D**) large virion-containing vacuoles (LVCVs) are large organelles with a smooth single limiting membrane and virions at their interior. Virions can be seen as invaginations of the limiting membrane (arrowheads). Black and white arrows indicate immature and mature virions, respectively; (**E**) large single-membrane compartments containing mature virion particles (asterisk), and filled with condensed membrane whorls and amorphous material/structures. White arrows indicate mature virions; (**F**) cytoplasmic inclusions composed of condensed tubular structures containing a dense inner core were observed connected to the endoplasmic reticulum (ER), which we named ER bodies (ERBs). From time to time, virus particles were observed in their interior (arrows). ER: endoplasmic reticulum; M: mitochondrion; N: nucleus; PM: plasma membrane. Scale bar: 250 nm.

As a first ultrastructural analysis, we compared the morphology of uninfected cells with that of those infected with PEDV for 72 h by EM in order to compile a repertoire of all the membranous rearrangements induced by this virus. We identified six different structures. The most abundant structure was the large double-membrane vesicles, which had a diameter of 230 ± 50 nm, contained coiled filaments, and were often in close proximity to each other (Figure 1A,B). These are the characteristic DMVs induced by *CoV* [12,17,23,24,25]. Numerous DMVs appeared to have inward invaginations of the limiting membrane, which have also previously been observed in specific cell types (Figure 1B, asterisks) [12,15]. In between and around the DMVs, we frequently observed a network of reticular inclusion, which had already been described in MHV- and SARS-CoV-infected cells as the CMs (Figure 1B) [12,15,17].

PEDV virions were also easily detected and appeared as spherical structures with a diameter range of 90 ± 20 nm (Figure 1C–F). Based on their morphology, PEDV particles could be grouped in two categories. The first were particles with an annular stained region under the external envelope (Figure 1C,D,F, black arrows). The second were smaller virions with a less circular and denser core, which was more markedly stained (Figure 1C–E, white arrows). These same phenotypical differences in viral particles have also been observed in TGEV-infected cells, another *α-CoV*, for which it was shown that the particles represent immature and mature virions, respectively [29]. PEDV viral particles were mainly present in the lumen of two morphologically distinguishable compartments, where they were assembling through inward budding at the limiting membrane (Figure 1C,D, arrowheads). One of these two compartments was what appeared to be an expanded rough ER with irregular shape, as the surface was decorated with ribosomes and they were often observed to be connected to an ER with normal morphology (Figure 1C). These structures have already been reported in cells infected by other *α-CoV* [23,29]. The other compartments were large vacuoles with a diameter of 865 ± 270 nm, limited by a single membrane, which have previously been named LVCVs (Figure 1D) [12,17,18,22,23,26,27,29,30]. Virions were also detected in large single-membrane vacuoles with a diameter of 800 ± 160 nm, which also contained compact membrane whorls and amorphous material (Figure 1E). Similar compartments, but smaller in size and without virions, were also observed in uninfected cells, indicating that they do not represent structures induced by PEDV. These characteristics suggested that these vacuoles could be endolysosomal compartments. This notion was confirmed by the immunogold labeling of cryo-sections obtained from PEDV-infected cells at 72 h p.i. using antibodies against CD63 and the transferrin receptor (TfR). These two marker proteins of the endolysosomal system [44] were present in these virion-containing vacuoles (Figure S1). Altogether, these characteristics indicate that these vacuoles are expanded endolysosomal compartments. Interestingly, both types of virions could be observed in ER and LVCVs, but only the smaller dark mature virions were found in the endolysosomal compartments.

Finally, we also detected large cytoplasmic inclusions with a length of about 910 ± 288 nm, which were characterized by a dense inner core with a geometrical appearance, a limiting membrane, and connections with the ER (Figure 1F). Virions could also be seen to form in those structures (Figure 1F, arrows), which have already been reported in cells infected by human coronaviral LINDER strain [45]. We named this new structure ER bodies (ERBs). We could not detect structures reminiscent of TBs and CMSs.

### 3.2. Time-Course PEDV Infection and Measurement of Cellular Lifecycle Parameters

To understand the relationship between the different membranous structures induced by PEDV and obtain insights into their role during infection, we subsequently infected cells with PEDV and examined them at the ultrastructural level in a time-course manner, at different timepoints between 0 h and 72 h p.i. We first measured, at each p.i. timepoint, important known parameters that reflect the viral lifecycle, i.e., infection efficiency, RNA replication/transcription, and secretion of progeny virus, to be able to correlate our EM analyses with the progression of *α-CoV* infection in host cells.

**Figure 2:**
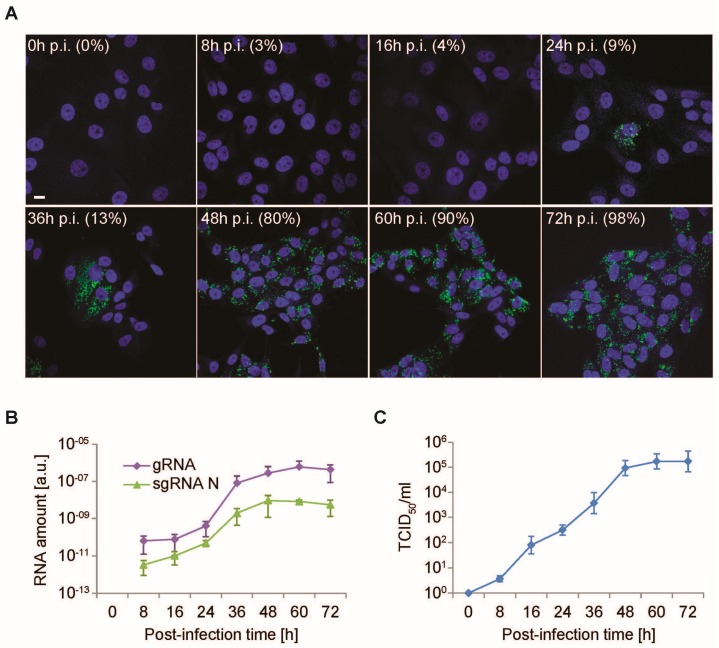
Time-course PEDV infection and measurement of cellular lifecycle parameters. Vero E6 cells were infected with PEDV (MOI = 1) prior to analyzing them and the cell culture supernatants at 0, 8, 16, 24, 36, 48, 60, and 72 h p.i. (**A**) Cells were processed for immunofluorescence using an anti-dsRNA antibody (green) to determine the number of PEDV-positive cells. The 4′,6-diamidino-2-phenylindole (DAPI) dye (blue) was used to stain the nuclei and determine the total number of cells. Scale bar: 10 µm. Quantification of the percentage of infected cells at each timepoint is indicated between brackets; (**B**) the total RNA was isolated from cells and the relative amount of sgRNA N and gRNA mRNAs was quantified by RT-PCR; (**C**) the production of the virus progeny was assessed by determining the virus titer of the cell culture supernatants by end point dilutions on Vero E6 cells, before calculating the 50% tissue culture infectious dose (TCID_50_)/mL. Error bars in (**B**,**C**) represent the standard deviation of three experiments.

Virus infection was examined at each timepoint by immunofluorescence using anti-dsRNA antibodies to indirectly monitor the progression of PEDV infection over time. dsRNA is an intermediate in *CoV* replication; it principally localizes inside the DMVs, and therefore can be used to specifically detect cells in which *CoV* are replicating [46]. As shown in Figure 2A, the dsRNA could be visualized from 24 h p.i., but the percentage of cells positive for this nucleic acid was still below 10% (Figure 2A), while it increased slightly to 13% at 36 h p.i. A very pronounced PEDV infection was detected at 48 and 72 h p.i., with 80% and almost 100%, respectively, of cells positive for dsRNA (Figure 2A).

Next, the amounts of both gRNA and the subgenomic RNA encoding for the structural N protein (sgRNA N) were determined at each timepoint by RT-PCR to assess the RNA replication/transcription of PEDV. Both gRNA and sgRNA N were already detected at 8 h p.i. and their amounts gradually increased until 48 h p.i., when they reached an expression plateau (Figure 2B).

Finally, the presence of infectious virions in the cell culture supernatants was determined using the TCID_50_/mL titration to monitor the assembly and release of PEDV. The presence of PEDV virions was first detected at 8 h p.i. and increased until 48 h p.i., when it reached a maximum (Figure 2C). This observation correlated with the analysis of viral RNA expression because, as expected, PEDV assembly and release follow intracellular PEDV replication.

Altogether, these measurements indicated that the PEDV lifecycle in Vero E6 cells progresses following the established dynamics of *CoV*, thereby confirming the use of this cell line as a model [40]. Moreover, these quantifications showed that PEDV starts to replicate, assemble, and egress in these cells around 8 h p.i., and the infection continuously increases until 48 h p.i. (Figure 2). At this timepoint, there is a dramatic augmentation in the number of infected cells, close to 100%, which coincides with an arrest in the augmentation of viral RNA synthesis.

### 3.3. Quantification of the PEDV-Induced Structures over the Course of an Infection

**Figure 3:**
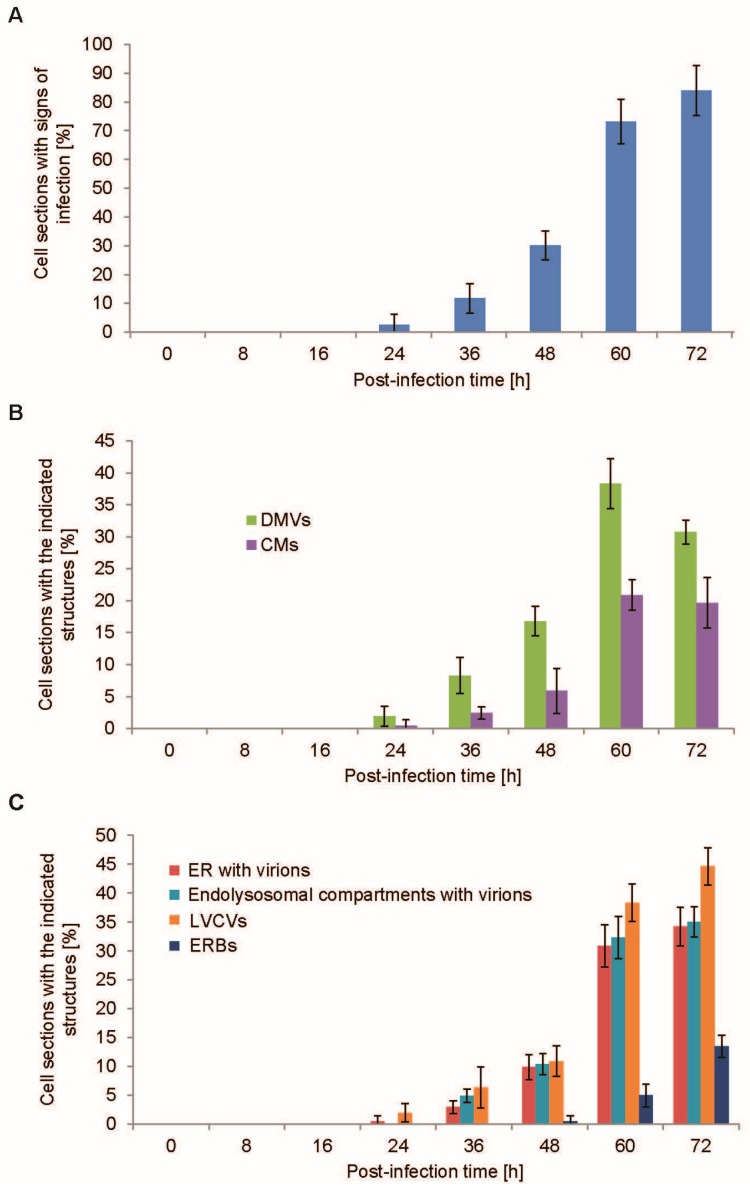
Quantitative analysis of the PEDV-induced structures over the course of the infection. (**A**) The EM preparations described in Figure 1 were used to count the number of cell sections containing at least one of the six PEDV-induced structures (Figure 1) to morphologically assess the proportion of infected cells; (**B**) the percentage of cells displaying DMVs and CMs was determined in the experiments shown in Figure 1; (**C**) the percentage of structures containing viral particles (i.e., virion-positive ER, virion-positive endolysosomal compartments, LVCVs, and ERBs) in the samples shown in Figure 1 was statistically evaluated. Error bars represent the standard deviation from four grids.

Next, we quantified the number of the PEDV-induced structures that we inventoried at 72 h p.i. (Figure 1), at each collected p.i. timepoint by conventional EM. We first morphologically determined the number of cell sections that showed visible signs of infection to see whether the changes observed at the ultrastructural level correlate with the measured infection parameters (Figure 2). To this end, the number of cell sections displaying at least one of the PEDV-induced structures detected at 72 h p.i. (Figure 1) was determined. At 24 h p.i., 2% of the cell sections started to show visible signs of infection, and this percentage gradually increased over time until it reached 84% at 72 h p.i. (Figure 3A). Importantly, the percentage of cell sections with visible signs of infection obtained from the EM analysis correlated well with the rest of the measured parameters (Figure 2), showing that the morphological examination of the cells is a reliable alternative approach to follow PEDV infection.

We next quantitatively analyzed the EM samples prepared at the different p.i. timepoints to understand the role of the PEDV-induced structures during an infection and to unravel their relationship. In particular, the percentage of cell profiles containing a specific structure was determined for each p.i. timepoint (Figure 3B,C). DMVs were one of the first structures to be detected, and they were observed in about 2% of cell sections at 24 h p.i. The number of cell sections positive for these vesicles gradually increased over time, reaching a maximum of 38% at 60 h p.i. (Figure 3B). Interestingly, the localization and morphology of the DMVs changed over the course of the PEDV infection (Figure S2). At early timepoints, i.e., 24 and 36 h p.i., DMVs had always a regular circular shape and they were distributed throughout the cytoplasm in small clusters of less than five DMVs. From 48 h p.i. onwards, DMVs became organized in larger clusters, with 10 or more vesicles, and mostly were found in the perinuclear region of the cell. The DMVs invaginations also became more pronounced from 48 h p.i. CMs were initially detected at 24 h p.i., but only in 0.5% of cells (Figure 3B and Figure S2). They became more apparent at 36 h p.i., reaching a plateau at 60 h p.i. The CMs were always found in between or around the cluster of DMVs. A dramatic change in the percentage of CMs was observed at 60 h p.i., increasing from 6% to 21%, when the DMVs also increased markedly. Overall, these data indicated that the CMs are structures that are functionally connected with DMVs, as previously reported [15].

Both LVCVs and virions-positive ER were detectable from 24 h p.i., and the percentage of cell sections with these structures increased over time (Figure 3C). Interestingly, their morphology changed with the progression of the infection. At earlier timepoints, from 24 to 48 h p.i., the number of virions per ER and LVCV section was less than five. At late timepoints, i.e., 60 and 72 h p.i., the virion-positive ER domains and LVCVs were larger, had less regular shapes, and contained more than 10 viral particles in their interior. Virion-positive endolysosomal compartments started to appear from 36 h p.i., and the number of virions found in their lumen increased over time. These observations suggested that the formation of LVCVs, virion-positive ER, and virion-positive endolysosomal compartments are probably induced by a higher production of virions in the cell.

ERBs started to become visible only at 48 h p.i. (Figure 3C). Initially, positive cell profiles displayed only one ERB, but from 60 h p.i. onwards we occasionally observed more than one ERB per cell section. We concluded that the ERBs are not required for the early steps of the PEDV lifecycle, but are rather the result of an advanced infection.

### 3.4. The Golgi Complex Undergoes Reorganization over the Course of a PEDV Infection

**Figure 4:**
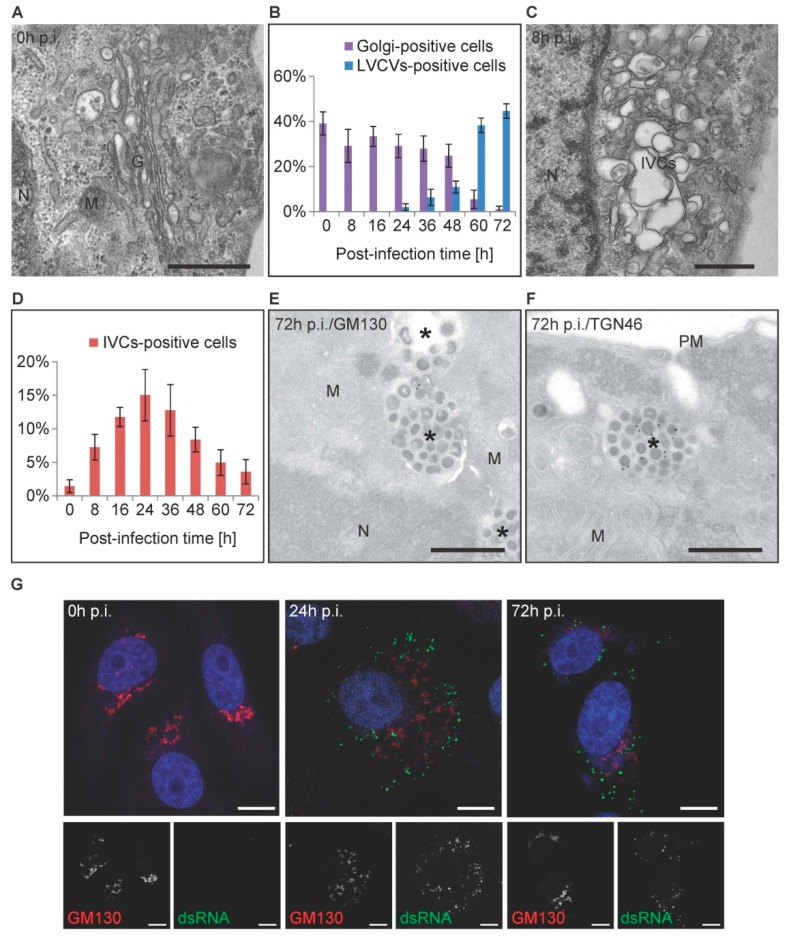
The Golgi complex undergoes reorganization over the course of a PEDV infection. Vero E6 cells infected with PEDV were collected at different p.i. timepoints, as described in the Materials and Methods section, before being processed for conventional EM (**A**–**D**), immuno-EM (**E**,**F**), and immunofluorescence (**G**). (**A**) A Golgi complex observed at 0 h p.i., which is typically composed of few long tubular stacks, with vesicles in their proximity; (**B**) the percentage of cell sections in the experiment shown in Figure 1 that displays a Golgi complex and/or an LVCV; (**C**) typical morphology of an irregular vesicle cluster (IVCs), which is a closely packed, irregular shaped, single-membrane vesicle with a light content; (**D**) the percentage of cell sections in the experiment shown in Figure 1 which are positive for IVCs. (E, F) PEDV induces the formation of LVCVs, which are positive for the Golgi stack and trans-Golgi protein markers GM130 (**E**) and TGN46 (**F**), respectively. Asterisks mark the LVCVs; (**G**) the subcellular distribution of GM130 (red) was examined by immunofluorescence over the course of a PEDV infection (green), at 0, 24, 48 and 72 h p.i. The anti-dsRNA staining was employed to identify infected cells and nuclei were stained with DAPI dye (blue). Representative immunofluorescence images were from three independent experiments. Error bars represent the standard deviation from three grids. G: Golgi; M: mitochondrion; N: nucleus; PM: plasma membrane. Black scale bar: 500 nm; white scale bar: 10 μm.

Our ultrastructural quantifications showed that the LVCVs become very prominent at 60 h p.i. (Figure 3C). It has previously been shown that LVCVs are expanded Golgi stacks [12,29]. Therefore, we decided to quantify at the ultrastructural level the number of cell sections displaying the presence of at least one Golgi complex over the course of the PEDV infection (Figure 4A,B). During the first 48 h after the inoculum, the number of cell sections displaying this organelle did not change noticeably (Figure 4B). The Golgi complex, however, was practically no more detectable with its known morphology (i.e., a series of adjacent stacks) from 60 h p.i., at the exact same timepoint when the LVCVs became prominent (Figure 4B). Interestingly, the percentage of cell sections with LVCVs at the late p.i. timepoints was similar to those of cell sections with Golgi prior to exposure to PEDV. Altogether, these data indicated that, as for MHV and TGEV [12,29], the LVCVs originate from ERGIC/Golgi, which probably expand as a consequence of a large local production of virions.

During the morphological analysis of the Golgi complex, we also observed another alteration of this compartment, which appeared as large clusters of single membrane vesicles with irregular contours and very variable lengths between 650 nm and 1.9 µm (Figure 4C). In approximately 20–50% of the cases, depending on the p.i. timepoint, these structures also appeared to contain a few Golgi-like stacks, and we therefore speculated that they originate from the Golgi complex (Figure S3A,B). Quantification of these structures revealed that they are not abundant, and the number of cell sections displaying them peaked at 24 h p.i. before decreasing (Figure 4D). These structures have not been detected in other *CoV* infections and therefore they could be specifically induced by PEDV. Alternatively, they could represent Vero E6 cell-specific Golgi complex alterations that take place when those cells are exposed to PEDV. We named those structures irregular vesicle clusters (IVCs).

Subsequently, we also examined whether the Golgi organization and subcellular distribution changes during the course of a PEDV infection by fluorescence microscopy. We inoculated cells with PEDV (MOI = 1) before analyzing them by immunofluorescence at 0, 24, and 72 h p.i. using antibodies against dsRNA and GM130, a Golgi marker protein. In non-infected cells, GM130-labelled Golgi localized perinuclearly (Figure 4G) [47]. GM130 lost its compact organization in infected cells, recognized by dsRNA staining, and became more scattered throughout the cytoplasm in numerous puncta at 24 h p.i. (Figure 4G). At 72 h p.i., the distribution of the GM130 signal changed again, forming cytoplasmic clusters, but those had a less compact shape compared to the ones observed in uninfected cells. We did not observe a colocalization between dsRNA and GM130 at any timepoint, indicating that the Golgi membranes very likely do not contribute to the establishment of the PEDV replication sites (Figure 4G). When the same samples were labeled with another Golgi protein marker, GIANTIN [48], and one for the trans-Golgi network, TGN46 [48], and analyzed by immunofluorescence, we detected the same reorganization of the Golgi in infected cells as that observed for GM130 (Figure S3C,D).

To demonstrate that LVCVs indeed originate from Golgi complexes, Vero E6 cells were infected with PEDV for 72 h before being processed for immuno-EM as described in the Materials and Methods section. Cryo-sections were subsequently immunogold labelled with anti-GM130 and anti-TGN46 antibodies. In uninfected cells, GM130 and TGN46 were exclusively localized to the Golgi complex (Figure S3E), where they were present in the stacks and trans-Golgi network, respectively, as expected. In contrast, GM130 and TGN46 labels were mainly found in LVCVs at 72 h p.i., confirming that these compartments have a Golgi origin (Figure 4E,F).

Altogether, the immunofluorescence results corroborate those obtained in EM analyses, and reveal that the Golgi complex undergoes a massive reorganization during PEDV infection.

### 3.5. PEDV Infections Involve ER Membrane Rearrangements in Vero Cells

**Figure 5:**
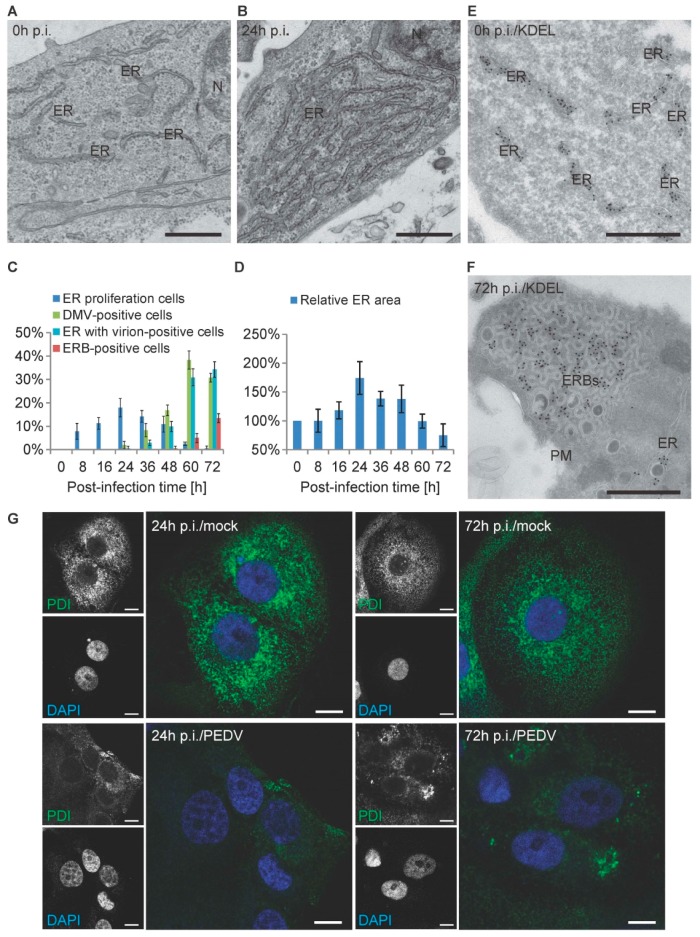
PEDV infection causes different ER membrane rearrangements. Vero E6 cells infected with PEDV were collected at different p.i. timepoints as described in the Materials and Methods section, before being processed for conventional EM (**A**–**C**), immuno- EM (**D**,**E**), and immunofluorescence (**F**). (**A**) ER observed at 0 h p.i., which typically appears as long dispersed tubular structures, with many ribosomes sitting on the limiting membrane; (**B**) representative morphology of proliferating ER in PEDV-infected cells, showing an arrangement of 5–10 layers of tubular rough ER; (**C**) the percentage of cell sections in the experiment shown in Figure 1 that displays ER proliferation, DMVs, CMs, virion-positive ER, and ERBs; (**D**) the ER surface area at each p.i. timepoint was determined using the point-hit method. The data are presented relative to the 0 h p.i. control; (**E**) morphology of the ER in non-infected cells labeled with an anti-KDEL antibody; (**F**) representative images of ERBs labeled with the anti-KDEL antibody; (**G**) the subcellular distribution of PDI (green), an ER protein marker, was examined by immunofluorescence with or without PEDV infection at 24 and 72 h p.i. Nuclei were stained with DAPI (blue). Representative immunofluorescence images were from three independent experiments. Error bars represent the standard deviation from three grids. M: mitochondrion; N: nucleus; PM: plasma membrane; ER: endoplasmic reticulum. Black scale bar, 500 nm; white scale bar, 10 μm.

The presence of virions in the ER and the formation of ERBs strongly suggested that there could also be a reorganization of the ER over the course of a PEDV infection. During the time-course EM analysis, we indeed observed that the ER proliferated over time, as numerous cross-sections of this organelle, often appearing as 5–10 adjacent ER tubules, were observed in the cytoplasm of infected cells (Figure 5A,B). The ER proliferation first appeared at 8 h and reached a peak at 24 h p.i., before becoming less prominent during the following hours of infection (Figure 5C). These changes in the ER area were confirmed by estimating the ER surface area using the point-hit method (Figure 5D). Interestingly, the ER proliferation alleviation starting from 24 h p.i. coincided with the emergence of ER-derived structures, such as DMVs, CMs, and virion-positive ER, in the infected cells (Figure 5C). Next, we confirmed that the ERBs as well as the virion-containing compartments seen in Figure 1C originate from ER by immuno-EM analysis using an anti-KDEL antibody (Figure 5E,F and Figure S4). Since the ERBs only appeared from 48 h p.i. and increased gradually afterwards (Figure 5C), this observation suggests that these structures are probably not functionally connected with ER proliferation.

To sustain the changes in the ER organization, we examined the ER organization during the course of PEDV infection by immunofluorescence. We inoculated cells with or without PEDV before analyzing them at 24 h and 72 h p.i. using antibodies against PDI, an ER resident protein [49]. In uninfected cells and at all timepoints, PDI localized in a large tubular network extending throughout the cytoplasm, as expected (Figure 5G) [50]. In infected cells, in contrast, PDI lost its homogenous distribution and concentrated perinuclearly at 24 h and 72 h p.i. This observation supports the EM analyses showing that ER undergoes a massive reorganization in PEDV-infected cells.

## 4. Discussion

The description of the membrane remodeling induced by PEDV in host cells remains poorly described despite the increasing veterinary importance of this virus [40,51]. This type of information is also scarce for *α-CoV* in general, as the infection of only two other viruses belonging to this genus, i.e., HCoV-NL36 and TGEV, have been characterized at the ultrastructural level so far [23,29,33]. HCoV-NL36 and TGEV virions have been observed in ER, Golgi, and LVCVs, while DMVs were solely detected in cells exposed to HCoV-NL36. Thus, although those studies have revealed some similarities between the host membrane rearrangements caused by *α-CoV* and members of the other *CoV* genera, a temporal and comprehensive description of all morphological alterations induced in host cells by *α-CoV* is still lacking. The limitation in the ultrastructural studies on HCoV-NL36 and TGEV has been the analysis of a single infection timepoint, which makes it difficult to obtain a complete repertoire of induced intracellular structures, because their frequency and morphology change over the course of an infection, as shown for MHV [12]. Therefore, we decided to take a time-course approach to examine PEDV infection in Vero E6 cells by qualitative and quantitative EM. In addition to structures like DMVs and LVCVs, already shown to be induced by *α-CoV*, this strategy allowed us to detect structures such as CMs, that have been reported for other *CoV*, as well as novel structures, such as IVCs and ERBs. It has recently been reported that PEDV triggers autophagy [52], but we did not observe evidence in our samples indicating an induction of this pathway. In particular, we did not detect autophagosomes, which are morphologically different than the observed DMVs. These latter are much smaller (180–280 versus 500–1500 nm [53]) and they do present cytoplasmic component in their interior.

Here, we report for the first time the presence of CMs in cells infected with an *α-CoV* (Figure 1B), which are morphologically similar to those generated in cells exposed to other *CoV* [15,17,24]. In contrast to what has been observed for MHV and SARS-CoV [12,15], the CMs appear at the same time as DMVs but with lower frequency (Figure 3B). We favor the idea that CMs could originate from the DMVs and could have a function in replication and transcription similar to these structures. In contrast to MHV- and SARS-CoV-infected cells [15,17], we did not observed continuity between these two structures using our EM approach.

It is known that ER is capable of increasing in size upon treatment with specific drugs or protein overexpression [54,55]. Interestingly, we saw a proliferation of the ER at the early stage of PEDV infection, from 8 h p.i., which peaked at 24 h p.i. just before the appearance of the DMVs and the virion-positive ER (Figure 5C). Considering the functional interrelationship between DMVs and ER [15,45,50], one hypothesis is that the ER proliferates to accommodate the increasing mass of viral proteins that will form the DMVs and luminal virions. Another hypothesis is that one or more PEDV proteins present some structural and/or functional characteristics that lead to a more pronounced ER stress response.

At the late stages of PEDV infection, we observed at low frequency a novel structure similar to that described in cells infected by human coronaviral LINDER strain [45], which we named ERB (Figure 1F). ERBs have a geometrical organization and are connected with the ER (Figure 1F). Their ER origin is also demonstrated by the fact that they are positive for the ER marker peptide KDEL (Figure 5D,E). These observations render the ERBs reminiscent of the TBs and CMSs induced by MHV and SARS-CoV [12,16,17,31]. TBs and CMSs also appear at the late stage of an infection and contain a single viral protein, which self-aggregates when massively overproduced. As a result, it is tempting to speculate that ERBs also represent aggregates formed by one or more PEDV proteins that are present in the ER in elevated amounts. The morphological differences between ERBs, TBs, and CMSs are probably due to a difference in the structural and self-aggregating properties of the proteins that lead to their formation.

We also identified a novel structure that we called IVCs, which we speculate to probably originate from the Golgi, because some of the IVCs appeared to contain stacks of this organelle (Figure S3A,B). Like the ER proliferation, IVCs also became apparent at the early stage of PEDV infection, at 8 h p.i., and became the most frequent at 24 h p.i. (Figure 4D). As a result, a hypothesis analogous to that drawn for the ER proliferation would be that IVCs are Golgi complexes that are expanding and/or becoming altered because of the increasing amounts of PEDV proteins. One could also imagine that IVCs are the precursors to LVCVs.

In our study, we observed virions in several compartments, i.e., ER, LVCVs, endolysosomal organelles, and very rarely in the ERBs (Figure 1C–F). However, we first detected them forming in both ER and LVCVs at the same timepoint (i.e., 24 h p.i.). This is in contrast with what has been reported for *CoV* belonging to other subfamilies, i.e., their viral particles assemble in the ERGIC/Golgi compartments [12,56], but also TGEV. The virions of this *α-CoV* start to form in the ERGIC/Golgi compartment and only at later timepoint in the ER [29]. The reason behind these differences might be due to differences between genera, viruses, and/or infected cell types. Nonetheless, our findings suggest that PEDV particles assemble in both the ER and Golgi complex. PEDV virions were also detected in what appear to be endolysosomal-like compartments from 36 h p.i., but, intriguingly, we never noted them assembling on the limiting membrane of these organelles. Altogether, these observations lead us to postulate that the endolysosomal compartments are not the site of viral particle assembling, but rather a location where excess virions are nonspecifically delivered, or that could eventually represent a transport for intermediates involved in the secretion of viral particles. Alternatively, the virions observed in the endolysosomal compartments could be secreted viral particles that are re-infecting cells through endocytosis. Very interestingly, we observed two types of virions appearing simultaneously (Figure 1C,D), which have been so far described exclusively in cells exposed to another *α-CoV*, TGEV, suggesting that this could be a unique characteristic of this *CoV* genus [29]. In the morphological study of TGEV-infected cells [29], smaller viral particles with denser cores were identified as mature virions, while larger annular ones were noted as the immature precursors. Our observations suggest that PEDV virion maturation does not occur in a specific organelle, as both types of viral particles were found to be present in the ER and LVCVs (Figure 1C,D), indicating the structural maturation of PEDV virion particles could happen in both the ER and LVCVs. This result is in part dissimilar from that obtained from the study of TGEV, where it was shown that virion maturation mainly takes place in the Golgi [29]. Again, this difference could be specific to the virus or the infected cell type. Another possibility is that some mature virions are retro-transported to the ER after maturation in the Golgi due to their local massive production. Interestingly, we also found that only mature virions are present in the compartments of the endolysosomal system (Figure 1E), further supporting one of our hypotheses made above.

In conclusion, our study has characterized and temporarily ordered the different membranous rearrangements induced by PEDV. This information paves the way for future investigations of the function of these structures, which is crucial to understanding PEDV and in general the *α-CoV* cellular lifecycle, and might eventually help to develop novel therapies against them.

## Supplementary Materials

The following are available online at www.mdpi.com/1999-4915/9/9/251/s1.
